# Identification and validation of key biomarkers associated with macrophages in nonalcoholic fatty liver disease based on hdWGCNA and machine learning

**DOI:** 10.18632/aging.205374

**Published:** 2023-12-21

**Authors:** Ruowen Li, Mingjian Zhao, Chengxu Miao, Xiaojia Shi, Jinghui Lu

**Affiliations:** 1 Department of General Surgery, Qilu Hospital, Cheeloo College of Medicine, Shandong University, Jinan 250012, Shandong Province, China; 2 School of Medicine, Cheeloo College of Medicine, Shandong University, Jinan 250012, Shandong Province, China

**Keywords:** NAFLD, hdWGCNA, machine learning, macrophage, biomarker

## Abstract

Background: NAFLD has attracted increasing attention because of its high prevalence and risk of progression to cirrhosis or even hepatocellular carcinoma. Therefore, research into the root causes and molecular indicators of NAFLD is crucial.

Methods: We analyzed scRNA-seq data and RNA-seq data from normal and NAFLD liver samples. We utilized hdWGCNA to find module-related genes associated with the phenotype. Multiple machine learning algorithms were used to validate the model diagnostics and further screen for genes that are characteristic of NAFLD. The NAFLD mouse model was constructed using the MCD diet to validate the diagnostic effect of the genes.

Results: We identified a specific macrophage population called NASH-macrophages by single-cell sequencing analysis. Cell communication analysis and Pseudo-time trajectory analysis revealed the specific role and temporal distribution of NASH-macrophages in NAFLD. The hdWGCNA screening yielded 30 genes associated with NASH-macrophages, and machine learning algorithms screened and obtained two genes characterizing NAFLD. The immune infiltration indicated that these genes were highly associated with macrophages. Notably, we verified by RT-qPCR, IHC, and WB that MAFB and CX3CR1 are highly expressed in the MCD mouse model and may play important roles.

Conclusions: Our study revealed a macrophage population that is closely associated with NAFLD. Using hdWGCNA analysis and multiple machine learning algorithms, we identified two NAFLD signature genes that are highly correlated with macrophages. Our findings may provide potential feature markers and therapeutic targets for NAFLD.

## INTRODUCTION

Non-alcoholic fatty liver disease (NAFLD) is a clinicopathologic syndrome characterized by the occurrence of steatosis in more than 5% of hepatocytes which is not caused by alcohol and other well-defined hepatic injury factors [1]. It includes simple steatosis, nonalcoholic steatohepatitis (NASH), cirrhosis, and hepatocellular carcinoma (HCC) [2]. With the rising number of obese people, NAFLD has become one of the leading causes of liver disease in the world, with about 100 million people worldwide suffering from the disease according to recent statistics [3]. If not diagnosed and treated in time, it will not only lead to liver disease disability, but also has a strong connection to the elevated incidence of metabolic syndrome, type 2 diabetes mellitus, atherosclerotic cardiovascular disease, and colorectal tumors [4]. In addition, there are no specific drugs that can reverse NAFLD, and the only treatment for end-stage liver disease is a liver transplant [5]. Thus, there is an urgent need to elucidate the intrinsic molecular mechanisms of NAFLD pathogenesis and to search for specific biomarkers to develop effective preventive and therapeutic approaches.

The pathology of NAFLD is a complex network of mechanisms involving multiple factors. It is significantly influenced by the increased infiltration of immune cell subsets, such as monocytes, T lymphocytes, and neutrophils, along with the activation of liver-resident cells, such as Kupffer Cells (KCs) or Hepatic Stellate Cells [6]. Macrophages can be categorized according to their origin into liver tissue resident macrophages (KCs) and monocyte-derived macrophages. Several studies have demonstrated that cytokines and chemokines released by KCs are critical in promoting chronic steatohepatitis [7, 8]. Depletion of KCs by using gadolinium chloride or clodronate liposomes prevented the progression of diet-induced steatosis and hepatic insulin resistance in rats [9]. In addition, it has been shown that monocyte-derived macrophages that can infiltrate the liver during the disease also play a vital role in NAFLD, and the reduction of infiltrating macrophages with specific drugs can inhibit hepatic steatosis and fibrosis [10].

**Figure 1:**
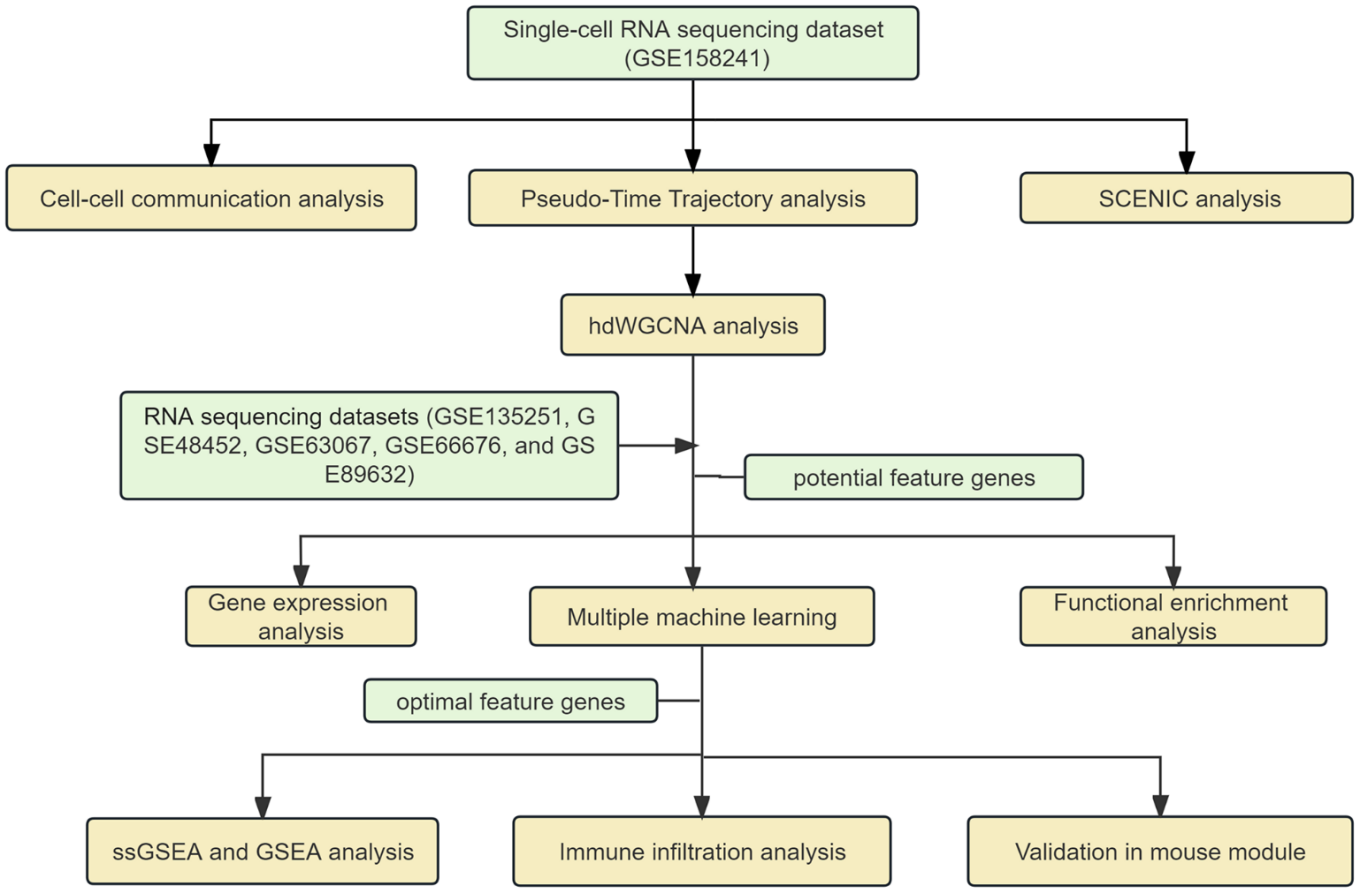
The flow chart of our analysis.

Over the past few years, the rapid development of single-cell transcriptomics has revolutionized the high-resolution analysis of cellular composition and heterogeneous cellular states, which has considerably contributed to our understanding of the composition of immune cells in liver tissues in NAFLD disease [11]. Weighted gene co-expression network analysis (WGCNA), an unbiased systems biology analysis method, aims to explore the co-expressed gene modules and identify core genes in the networks, whereas it can only be used for bulk RNA sequencing (RNA-seq) data [12]. Unlike WGCNA, the high-dimensional weighted correlation network analysis (hdWGNCA) could constitute an integrated functional framework for co-expression networks based on single-cell RNA sequencing (scRNA-seq) data [13]. Previous studies have not delved into the characteristic markers of NAFLD at the single-cell level [14]. In our investigation, we coordinated scRNA-seq data and RNA-seq datasets to screen key signature genes contributing to NAFLD diagnosis by hdWGCNA and multiple machine learning algorithms, which may contribute to the early diagnosis and treatment of NAFLD (Figure 1).

## MATERIALS AND METHODS

### Data acquisition

We obtained a NAFLD single-cell RNA sequencing dataset (GSE158241)and five NAFLD RNA sequencing datasets (GSE135251 [15], GSE48452 [16], GSE63067 [17], GSE66676 [18], and GSE89632 [19]) from the Gene Expression Omnibus (GEO) database (http://www.ncbi.nlm.nih.gov/geo). For the RNA-seq datasets, we firstly utilized the “GEOquery” software package to download the data and clinical information [20]. Secondly, the “SVA” package was used for background correction, normalization, and log2 transformation [21]. When multiple probes of the platform identified the same gene, the average value was taken as its expression. Subsequently, the GSE63067 and GSE89632 datasets were merged as the experimental group (31 normal and 50 NAFLD samples), while the GSE48452, GSE66676, and GSE13251 datasets were merged for further validation (85 normal and 271 NAFLD samples). The “SVA” package was used to eliminate batch effects after merging datasets [21]. Table 1 provides information on these six datasets’ elements.

**Table 1:** The basic information of datasets.

**Dataset**	**Year**	**Species**	**NAFLD**	**Normal**	**Platform**	**Data type**	**Team**
GSE158241	2020	Mus musculus	2	4	GPL19057	scRNA-seq	Rigbolt KG, et al.
GSE135251	2020	Homo sapiens	206	10	GPL18573	Bulk RNA-seq	Govaere O, et al.
GSE48452	2013	Homo sapiens	32	41	GPL11532	Microarray	Jochen H, et al.
GSE63067	2014	Homo sapiens	11	7	GPL570	Microarray	Frades I
GSE66676	2017	Homo sapiens	33	34	GPL6244	Microarray	Xanthakos S, et al.
GSE89632	2016	Homo sapiens	39	24	GPL14951	Microarray	Allard JP, et al.

### Preprocessing of single-cell RNA sequencing data

Using the “Seurat” (4.1.0) package [22], we created Seurat objects based on the single-cell transcriptomic expression matrices of overall and individual cell types. We identified cells expressing over 200 but no more than 2500 RNA features. Additionally, 10% of mitochondrial RNA was set as a threshold for normalizing the scRNA-seq data. The batch effect of the samples was eliminated by the “harmony” functions. Furthermore, we used the “ScaleData” and “RunPCA” functions to determine the number of principal components (PCs) based on the Seurat object. We adjusted the number of PCs to 12 to generate cell clusters and then visualized them using the “UMAP” plot. To annotate the cell clusters, we performed unsupervised clustering using the “FindClusters” and “FindNeighbors” functions. The clustering results were obtained with the clearest resolution when the resolution was set to 0.5. Subsequently, we used the “SingleR” package (v 1.4.1) [23] for automated cell type annotation based on the marker genes of each cluster. Finally, we selected macrophages for principal component analysis (PCA) to identify distinct macrophage subtypes.

### High-dimensional weighted correlation network analysis

We employed the high-dimensional weighted gene co-expression network analysis (hdWGCNA) to construct a co-expression network based on single-cell level data using the “hdWGCNA” package [13]. First, we input the genes expressed in at least 5% of the cells and use the “MetacellsByGroups” function to construct the metacell gene expression matrix. Then, the “TestSoftPowers” function is used to determine the soft power. The “ConstructNetwork” function is used to build the co-expression network. All analyses are performed according to the official standard procedure as described in https://smorabit.github.io/hdWGCNA/articles/basic_tutorial.html.

### Cell-cell communication analysis

The “CellChat” package provides an effective analysis tool for studying the interactions and communications between cells [24]. We used the “CellChat” package to infer important biological interactions between cells in the liver, and calculated the probability values and significance of these interactions. Circle plots and bubble plots were used to visualize the relationships and importance between cells.

### Pseudo-time trajectory and SCENIC analysis

Monocle2 algorithm (version 2.22.0) [25] can infer the temporal development and differentiation trajectories of cells, as well as explore the transition relationships between cell states. In our study, we employed monocle2 (v2.18.0) for trajectory analysis to further investigate the differentiation process of macrophages in the liver. Additionally, Single-cell regulatory network interference and clustering (SCENIC, version 1.2.4) was employed on all single cells to unveil the regulatory relationships between transcription factors (TFs) and target genes [26]. The “limma” package was utilized to calculate significantly distinctly expressed regulators, with a statistical significance level set at p < 0.05.

### Functional enrichment analysis and GSVA analysis

To test the gene expression level in RNA-seq datasets, we employed the “limma” package in R to compare the expression differences of the feature genes between NAFLD samples and control samples. And to investigate the functional abundance of the potential feature genes, we used the “clusterProfiler” package (v4.0) for Gene Ontology (GO) and Kyoto Encyclopedia of Genes and Genomes (KEGG) pathway enrichment analysis. Additionally, the gene set variation analysis (GSVA) algorithm was applied to explore the activity variations of KEGG pathways in the optimal feature genes. The statistical significance level was set at p < 0.05.

### Building the machine learning model

To assess the diagnostic capability of the potential feature genes identified by hdWGCNA, we employed seven machine learning algorithms to build models using the mlr3verse (version 0.2.7) package in R(https://CRAN.R-project.org/package=mlr3verse). The predictive performance of the seven models was evaluated using the AUC values obtained from ROC analysis in the training set and validation set. To further select the optimal feature genes, we applied three machine learning algorithms (LASSO, SVM-REF, and RF) to predict disease status and identify important prognostic variables. For RF analysis, we used the “randomForest” package and the “caret” package in R [27] to determine gene importance, with a threshold set at an importance score greater than 2 [28]. We utilized the “glmnet” package in R [29] to perform LASSO logistic regression analysis with the value of lambda min. An SVM classifier was created using the “e1071” package in R [30]. Next, the effectiveness of the optimal feature genes was thoroughly evaluated in the training and validation sets. The expression levels of the optimal feature genes in NAFLD tissues and control tissues were compared with the Wilcoxon rank-sum test. The predictive ability of the optimal feature genes was evaluated using receiver operating characteristic (ROC) analysis, and the area under the ROC curve (AUC) values were assessed. The statistical significance level was set at p < 0.05.

### Immune infiltration analysis

We utilized the CIBERSORT analysis technique [31] to evaluate the immune infiltration patterns in NAFLD samples and normal samples. In this analysis, the parameter “PERM” was set to 1000 and a significance threshold of p<0.05 was applied. The “pheatmap” package in R was employed to generate a heatmap that displays the 22 immune cell types, while the “vioplot” package was used to create boxplots illustrating their abundance. To assess the differences in immune cell proportions, we conducted Wilcoxon rank-sum tests, considering p<0.05 as statistically significant.

### Single-sample gene set enrichment analysis (ssGSEA) and gene set enrichment analysis (GSEA)

In order to gain a more comprehensive understanding of the activation status of the gene sets under investigation, we utilized the single-sample Gene Set Enrichment Analysis (ssGSEA) algorithm [21] to assess the relative levels of 50 hallmark gene sets (h.all.v7.5.1.symbols.gmt) in control and NAFLD samples. Moreover, we conducted Spearman correlation analysis to determine the associations between these 50 hallmark gene sets and the top feature genes. Additionally, to delve into the biological significance of the top feature genes, we performed GSEA using the “c2.cp.kegg.v11.0.symbols” gene set from the Molecular Signatures Database.

### Animal model construction

We purchased Ten C57BL/6 mice (males) aged 6 weeks from GemPharmatech (Nanjing, China). All mice were fed at room temperature and under standard light conditions. After 7 days of acclimatization feeding, the mice were randomly divided into 2 groups. The control group was fed chow diet, and the experimental group was fed a methionine-choline deficient (MCD) diet without any additional intervention. After four weeks, peripheral blood of the mice was collected and centrifuged to obtain serum. The levels of alanine aminotransferase (ALT) and aspartate aminotransferase (AST) in serum were determined by Roche Cobas test. Fresh livers were exercised and weighed, and portions of the livers were taken for triglyceride assay. After being fixed in 10% formalin, partial liver samples were dehydrated in a series of progressively stronger alcohol washing solutions. Following xylene cleaning, tissues were embedded in paraffin. Sections were roughly 3μm thick and stained with hematoxylin and eosin (H&E). For Oil Red O staining, the sections were initially incubated in 60% isopropanol, followed by staining with an Oil Red O staining solution and a second incubation in 60% isopropanol. All procedures were conducted in accordance with the guidelines of the Institute for the Study of Animals.

### RT-qPCR, WB, and IHC

Total RNA was isolated from mouse liver tissue and cDNA was synthesized using HiScript II Reverse Transcriptase (Vazyme, Nanjing, China). Real-time quantitative polymerase chain reaction (RT-qPCR) analysis was performed using SYBR Green mixture (Vazyme Biotech, Q711). We set actin as the reference gene for each sample. All primers were purchased from Sangon Biotech (Shanghai, China). The primers are given in Supplementary Table 1. For western blot (WB) analysis, total proteins were extracted from the liver samples with lysis buffer (ThermoFisher, 78443) containing 1% PMSF. Proteins (15μg) of each sample were loaded onto a gradient SDS-PAGE. Proteins were then transferred to a PVDF membrane. After blocking with 1% bovine serum albumin (Sigma, A7030), the primary antibodies were used for detection: MAFB (TD8895, 1:2000) (Abmart), CX3CR1 (13885-1-AP, 1:1000) (Proteintech) and Actin (66009-1-Ig, 1. 10000). Tissue levels of MAFB and CX3CR1 were also analyzed by immunohistochemistry (IHC) using MAFB (TD8895, 1:500) (Abmart) and CX3CR1 (13885-1-AP, 1:200) (Proteintech) according to standard protocols.

## RESULTS

### Single-cell RNA sequencing quality control and cell annotation

**Figure 2:**
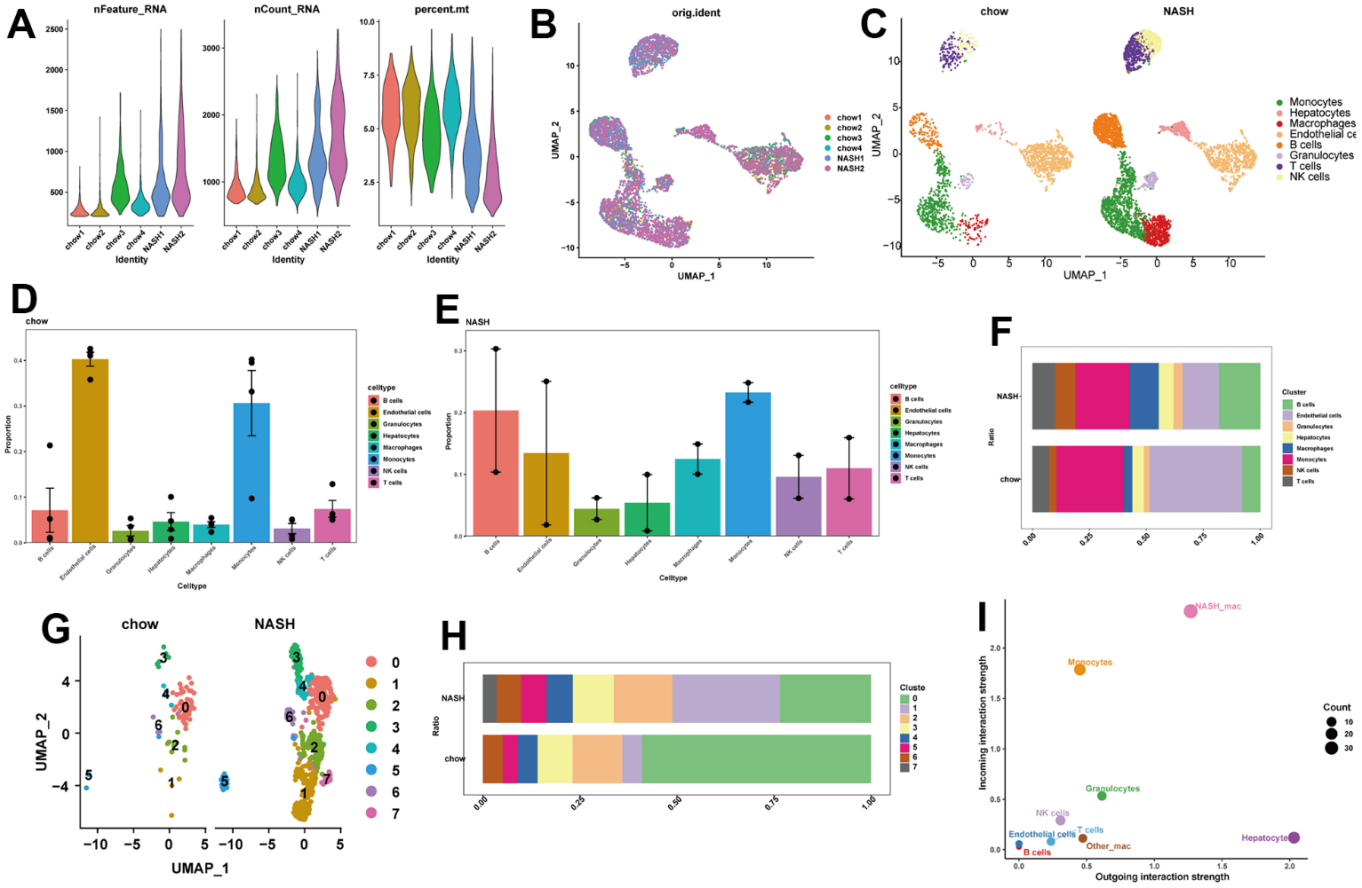
**Single-cell analysis of cell proportion of NAFLD.** (**A**) The features, counts, and percentages of mitochondrial genes in each of the analyzed samples after quality control. (**B**) The elimination of batch effect. (**C**) UMAP plot visualizes the distribution of eight cell types in control and NASH mouse livers. (**D**) Bar plot indicating the cell proportion of all eight cell types in liver of a normal chow diet mice. (**E**) Bar plot indicating the cell proportion of all eight cell types in liver of NASH mice. (**F**) Cell fraction distribution differences between NASH and normal. (**G**) UMAP plot showing the distribution of different clusters of macrophages in livers. (**H**) Bar plot indicating the proportion of seven macrophage clusters in liver of control and NASH mice. (**I**) Scatter plot indicating the incoming and outgoing interaction strength of the cells.

We initially obtained a total of 6888 cells from a scRNA-seq data set containing the livers of two NASH mice and four normal mice. Following quality control and removal of batch effects, a total of 6875 cells were used for single-cell clustering analysis (Figure 2A, 2B and Supplementary Figure 1A–1D). After cell annotation, we could observe 8 distinct cell types on the UMAP plot, including hepatocytes, epithelial cells, endothelial cells, T cells, NK cells, macrophages, granulocytes, and B cells (Figure 2C). We then analyzed the proportions of different cell types in NASH and normal mouse livers (Figure 2D, 2E). Interestingly, macrophages, B cells, and NK cells showed increased proportions in NASH compared to the normal group (Figure 2F). Given the crucial role of macrophages in the liver, we selected macrophages for further analysis. After clustering the macrophages, we identified seven subclusters of macrophages. Notably, subcluster 1 was significantly more prevalent in the NASH group, and subcluster 7 was specific to NASH (Figure 2G, 2H). Therefore, we labeled subcluster 1 and subcluster 7 as NASH-macrophages. Next, we performed cell-to-cell communication analysis between different cells in the liver. The results revealed that NASH-macrophages exhibited highly active signaling communication with other cells, while the other-macrophages had rarely communicated with other cells. (Figure 2I). Of interest, NASH-macrophages received more signals than other cells, and hepatocytes were identified as the strongest senders (Figure 2I). This indicates that NASH-macrophages may play a critical role in the progression of NAFLD.

### Screening for modules representing NASH-macrophages by hdWGCNA

**Figure 3:**
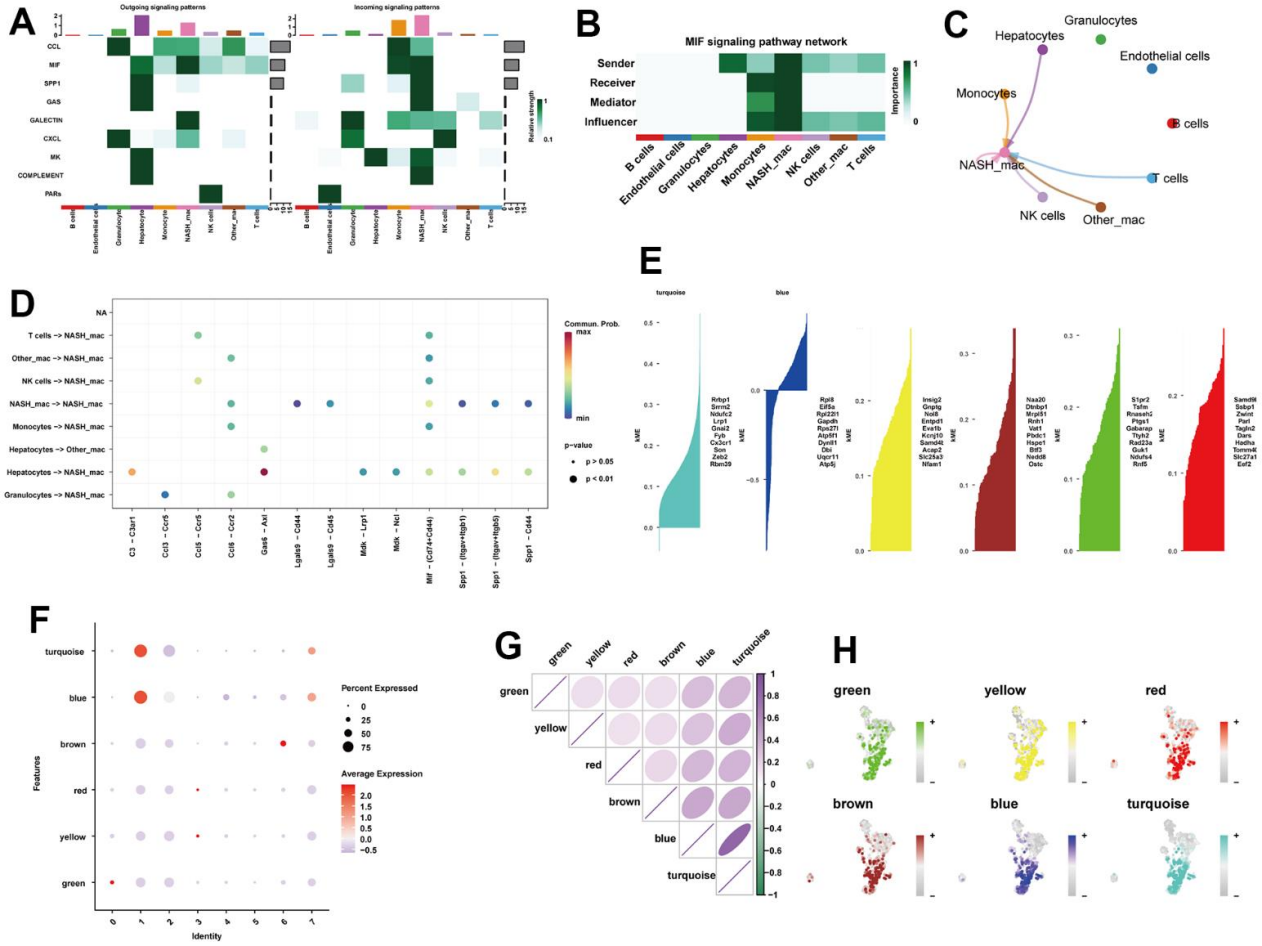
**Identification of the crucial modules related to NASH-macrophages by hdWGCNA.** (**A**) The dot plot showing the comparison of outgoing and incoming signaling patterns. (**B**) Heatmap showing the relative importance of each cell group in the MIF signaling network. (**C**) Circle plot showing the communication strength between interacting cells in the MIF signaling network. (**D**) Bubble plot showing the significant ligand-receptor pairs between cells. (**E**) Six gene modules were obtained and the top ten hub genes were presented according to the hdWGCNA pipeline. (**F**) Module activities in different macrophage clusters. (**G**) Correlation analysis between different models. (**H**) UMAP plots illustrating the distribution of each module.

Cell communication detected a total of 9 significant pathways, including CCL, MIF, SPP1, GAS, GALECTIN, CXCL, MK, COMPLEMENT, and PARs (Figure 3A). Of note, the macrophage migration inhibitory factor (MIF) signaling pathway exhibited great strength in both incoming and outgoing signal patterns of NASH-macrophages (Figure 3A). Within the MIF signaling pathway, NASH-macrophages were found to be the strongest sender, receiver, mediator, and influencer (Figure 3B, 3C). Ligand-receptor analysis indicated that Gas6-Axl was significantly activated in the paracrine signaling from hepatocytes to NASH-macrophages (Figure 3D). In view of the critical role of macrophages, we explored the hdWGCNA analysis to identify potential markers of macrophages. After setting the soft threshold to eight, we identified six modules (Supplementary Figure 2). As shown in Figure 3E, six gene modules were obtained, and the top 10 most influential genes were listed according to the hdWGCNA. Of interest, we found that the turquoise and blue modules were greatly expressed in subcluster 1 and subcluster 7 macrophages (Figure 3F). Additionally, the blue module exhibited a strong positive correlation within turquoise module (Figure 3G). Moreover, UMAP plots illustrated the distribution of the turquoise and blue modules in macrophages, which extremely overlapped with subcluster 1 and subcluster 7 macrophages (Figure 3H). Therefore, we proposed that the turquoise and blue modules may represent characteristics of NASH-macrophages. The top 20 genes from each of the turquoise and blue modules were considered as potential feature biomarkers of NAFLD.

### Pseudo-time analysis and transcription factor prediction

**Figure 4:**
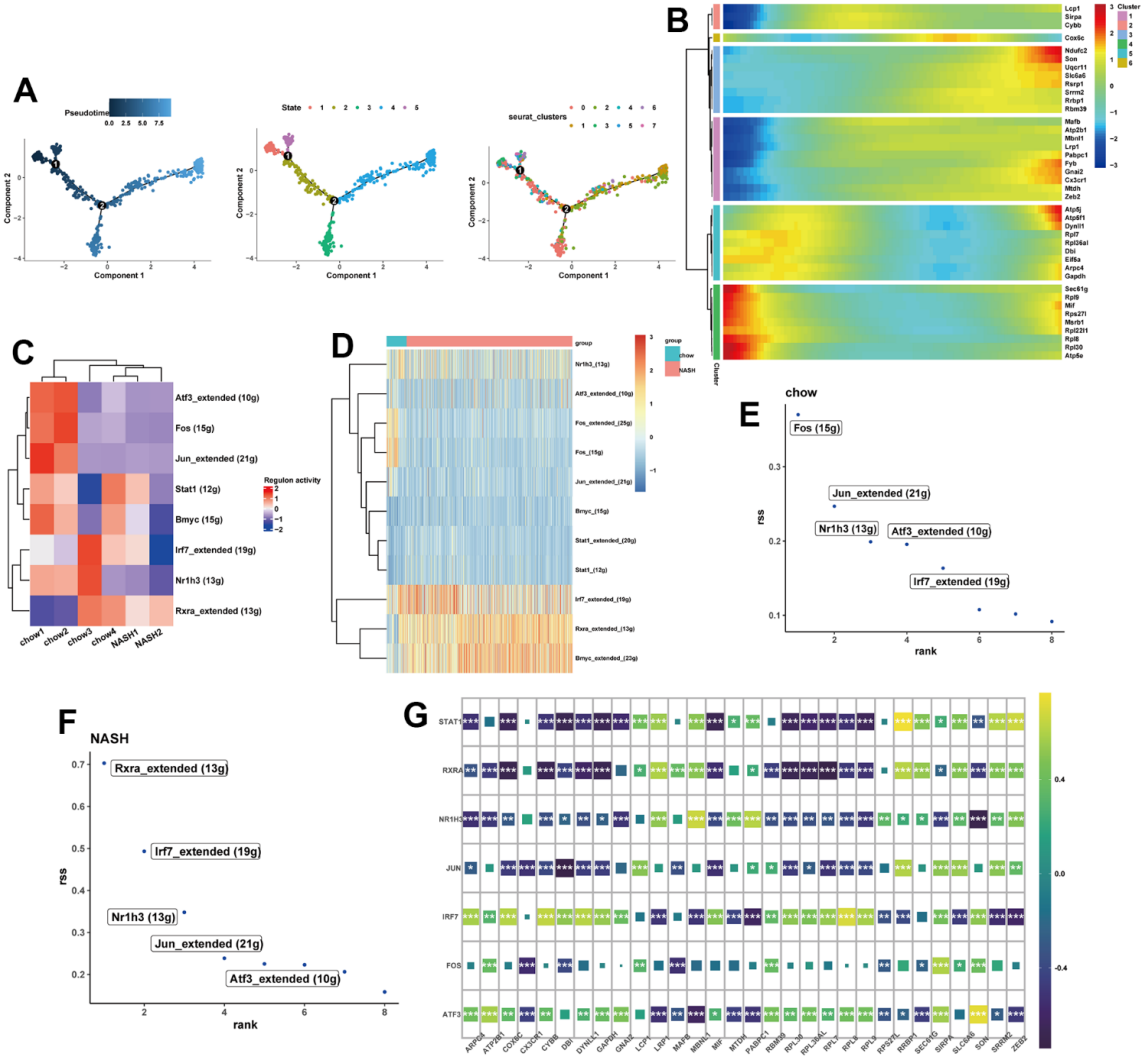
**Pseudo-Time trajectory and SCENIC analysis.** (**A**) pseudo-time distribution of the different macrophage subtypes. (**B**) Heatmap showing the change of potential feature genes in pseudo-time developmental trajectories. (**C**) Heatmap of RAS activity of transcription factors (TFs) in each sample, with negative correlations in blue and positive correlations in red. (**D**) Heatmap of the area under the curve (AUC) scores of TFs in each group. (**E**, **F**) Ranking of TFs in NASH and normal samples calculated by the RSS specificity score. (**G**) The correlation between SCENIC-identified TFs and 30 potential feature genes.

To determine the transcriptional features of macrophage development at different stages, we performed a pseudo-time analysis. Cells with similar states are grouped, and branch points separate cells into different states. Notably, cluster 1 and cluster 7 macrophages were mainly located at the end of the pseudo-time trajectory (Figure 4A). We intersected genes from the turquoise and blue modules with human transcriptional genes, resulting in 30 potential feature genes. Besides, the changes of potential feature genes in the differentiation were detected based on the gene expression levels in different subclusters of macrophages (Figure 4B). In order to further explore the transcriptional regulatory network underlying NASH, we subsequently used SCENIC algorithm to infer the transcription factors (TFs) behind NASH disease. SCENIC analysis revealed that certain TFs exhibited distinct activation and deactivation patterns across different samples (Figure 4C). We then detected 3 upregulated TFs and 8 downregulated TFs in the NASH group (Figure 4D). Additionally, a regulon specificity score (RSS) was defined based on Jensen-Shannon divergence for each group [32]. In the normal group, the transcription factor with the highest score was identified as Fos, while the RXRa ranked first in the NASH group (Figure 4E, 4F). In addition, transcription factors were closely related to potential feature genes (Figure 4G).

### Expression validation and functional enrichment of potential feature genes

**Figure 5:**
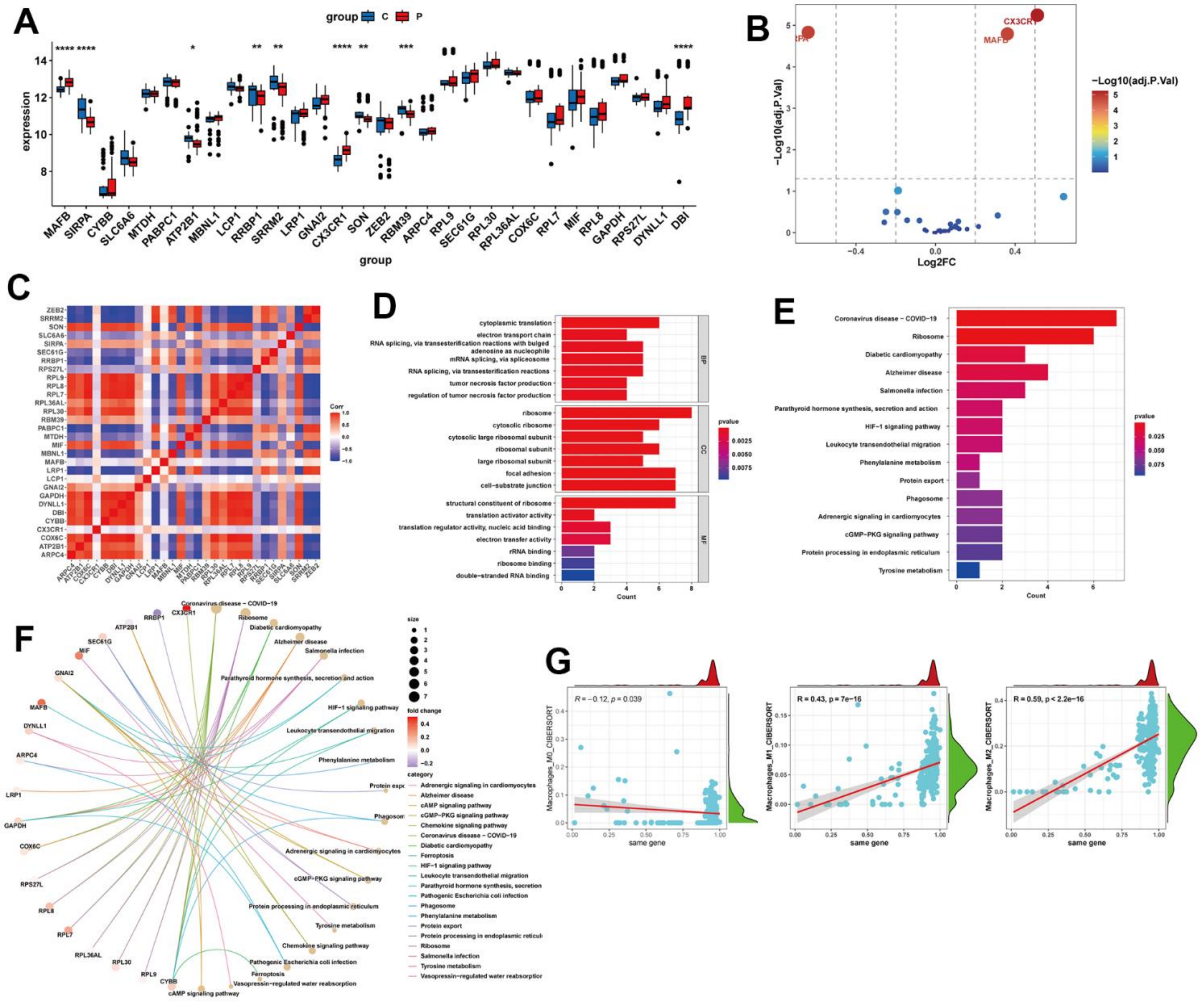
**Expression analysis and functional enrichment of potential feature genes.** (**A**, **B**) Expression analysis of potential feature genes between NAFLD and normal samples. (**C**) Correlation analysis between potential feature genes. (**D**) Enrichment analysis of potential feature genes using Gene Ontology (GO). BP, biological process; CC, cellular component; MF, molecular function. (**E**, **F**) Enrichment analysis of potential feature genes using the Kyoto Encyclopedia of Genes and Genomes (KEGG). (**G**) Correlation analysis between potential feature genes and M0, M1, and M2 macrophages. *P < 0.05, **P < 0.01, ***P < 0.001, ****P < 0.0001.

Next, we analyzed the expression levels of these 30 potential feature genes in the livers of normal individuals and NAFLD patients using RNA-seq data. We found that SIRPA, ATP2B1, RRBP1, SRRM2, SON, and RBM39 were significantly downregulated in NAFLD samples, while MAFB, CX3CR1, and DBI were significantly upgraded (Figure 5A). Among all the differentially expressed genes, CX3CR1, SIRPA, and MAFB showed the largest differences (Figure 5B). And these 30 genes exhibited close associations with each other (Figure 5C). In terms of GO enrichment analysis, the potential feature genes were enriched in biological processes (BP), such as Cytoplasmic translation, RNA splicing, via transesterification reactions with bulged adenosine as nucleophile, mRNA splicing, via spliceosome, RNA splicing, via transesterification reactions; and cellular components (CC), such as Ribosome, Focal adhesion, Cell-substrate junction; and molecular functions (MF), such as Structural constituent of ribosome (Figure 5D). In the KEGG enrichment analysis, these genes were significantly enriched in pathways related to Coronavirus disease-COVID-19, Ribosome, and Alzheimer’s disease (Figure 5E, 5F). We also analyzed the overall relationship of the potential feature genes as a whole and their associations with immune cell infiltration. The results showed a significant association between the potential feature genes and M0, M1, and M2 macrophage infiltration, with a negative correlation with M0 and positive correlations with M2 and M1 (Figure 5G).

### Identifying optimal feature genes of NAFLD by machine learning

**Figure 6:**
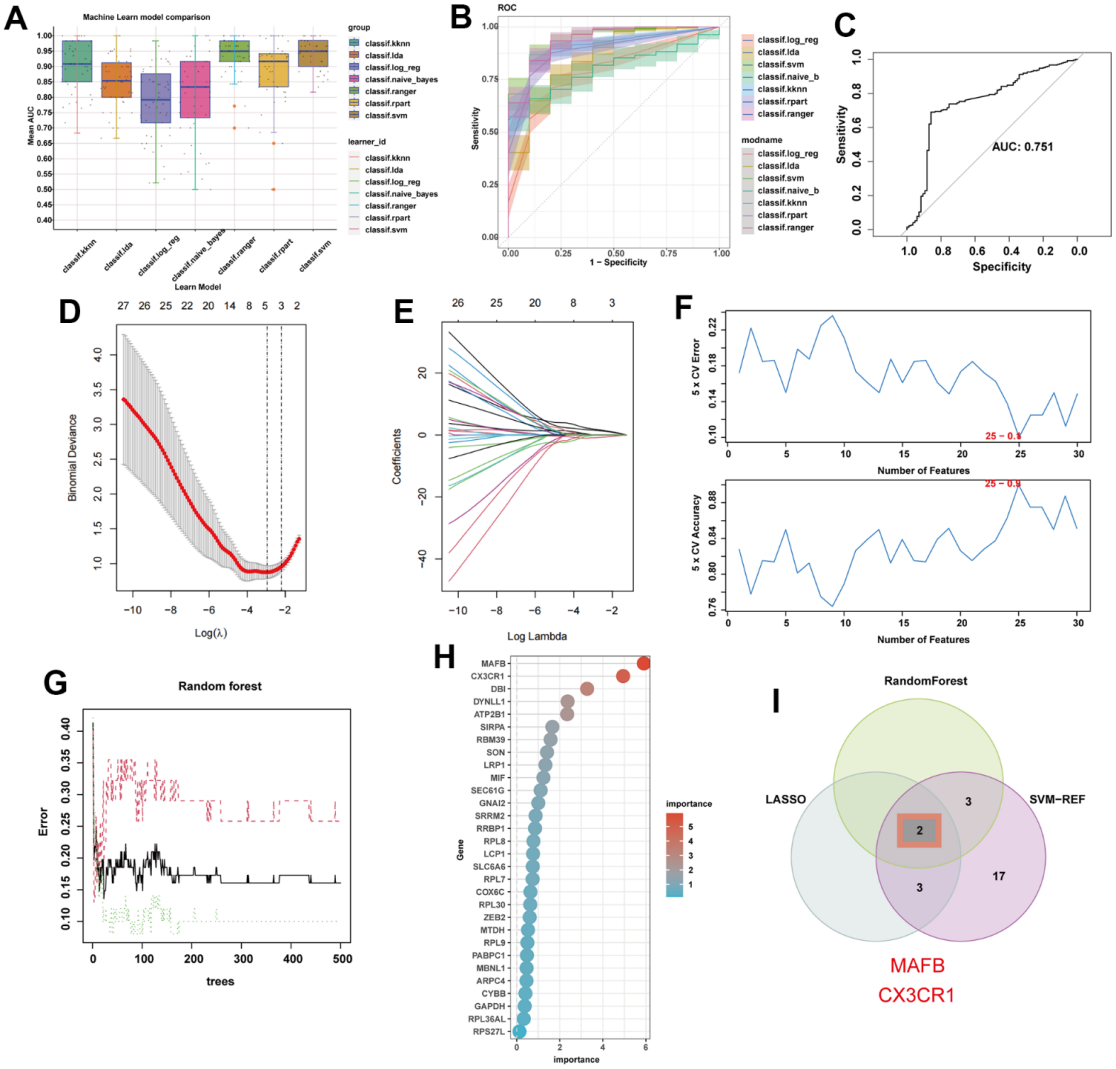
**Machine learning identifies optimal feature genes of NAFLD.** (**A**) Seven machine learning algorithms were utilized for model construction. (**B**) The ROC values of all seven algorithms in the training group. (**C**) The ROC scores of the SVM model were presented in the test group. (**D**) Lasso algorithm for selection features. (**E**) Coefficient changes of the selected features using lasso algorithm. (**F**) The SVM algorithm was used to further candidate optimal feature genes with the highest accuracy (the lower) and lowest error (the upper) obtained in the curves. The x-axis represents the number of feature selections, and the y-axis indicates the prediction accuracy. (**G**) The impact of the number of decision trees on the error rate was examined. The x-axis represents the number of decision trees, while the y-axis indicates the error rate. (**H**) The relative importance of potential feature genes was calculated in random forest (Top 5 genes’ importance > 2). (**I**) Venn diagram showing the overlap between the three algorithms.

Based on the potential feature genes, seven machine learning algorithms were applied to construct models and evaluate diagnostic performance (Figure 6A). It is worth noting that the support vector machine (SVM) algorithm showed the best performance in the training set (Figure 6B), with an AUC of 0.751 in the external validation set (Figure 6C). Next, we selected three machine learning algorithms to further screen for key feature genes of NAFLD. LASSO regression was performed using the aforementioned 30 genes as input, resulting in 5 genes (Figure 6D, 6E). We filtered 25 genes using the SVM algorithm (Figure 6F) and 5 genes using the Random Forest algorithm (Figure 6G, 6H). Finally, we exploit the intersection of these gene sets to identify the optimal diagnostic genes for NAFLD: MAFB and CX3CR1 (Figure 6I).

**Figure 7:**
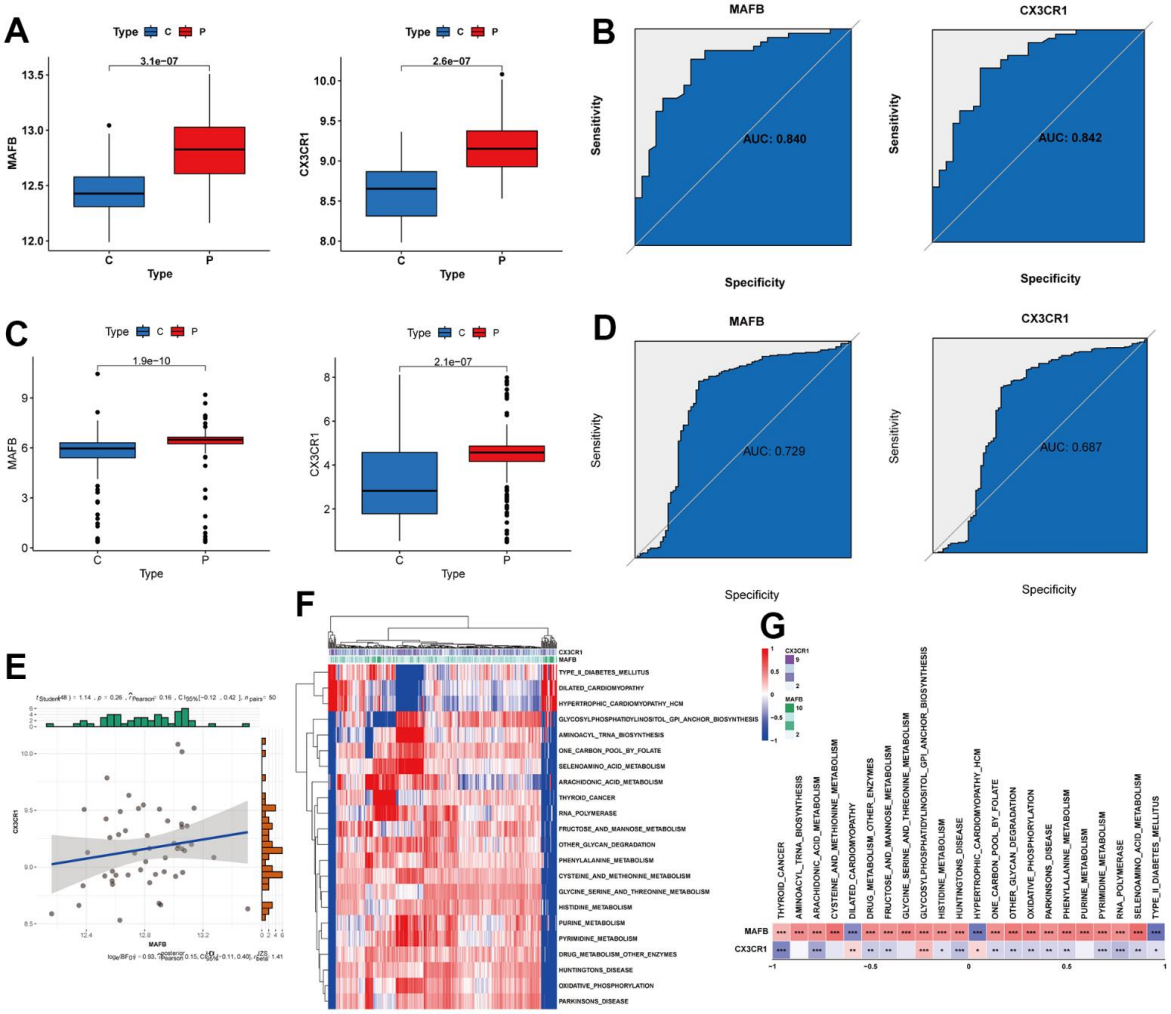
**Verification of expression and diagnostic efficacy for optimal feature genes.** (**A**) MAFB and CX3CR1 mRNA expression in the training group. (**B**) ROC curves of MAFB and CX3CR1 in the training group. (**C**) MAFB and CX3CR1 mRNA expression in the testing group. (**D**) ROC curves of MAFB and CX3CR1 in the testing group. (**E**) Correlation analysis between MAFB and CX3CR1. (**F**) Heatmap showing the scores of KEGG pathways in the optimal feature genes as calculated by GSVA. (**G**) Heatmap showing the correlation between the gene pathway and optimal feature genes. *P < 0.05, **P < 0.01, ***P < 0.001.

To validate the performance of the two key genes, we split the RNA-seq dataset into a training and validation set. Both MAFB and CX3CR1 consistently showed higher expression in NAFLD liver samples in both sets (Figure 7A, 7C). We assessed the diagnostic performance of these two feature genes using the ROC analyses. In the training set, MAFB and CXCR1 had AUC values of 0.840 and 0.842, respectively (Figure 7B). In the validation set, the AUC values were 0.729 for MAFB and 0.687 for CX3CR1 (Figure 7D). Furthermore, the expression of MAFB was positively linked with that of CX3CR1 (Figure 7E). Additionally, we performed pathway analysis using GSVA to identify KEGG pathways potentially associated with these two genes. As shown in Figure 7F, 7G, a total of 22 pathways were significantly associated with these two genes, including Arachidonic acid metabolism, Histidine-metabolism, Oxidative phosphorylation, Phenylalanine metabolism, Pyrimidine Metabolism, and so on.

### Immune infiltration analysis

**Figure 8:**
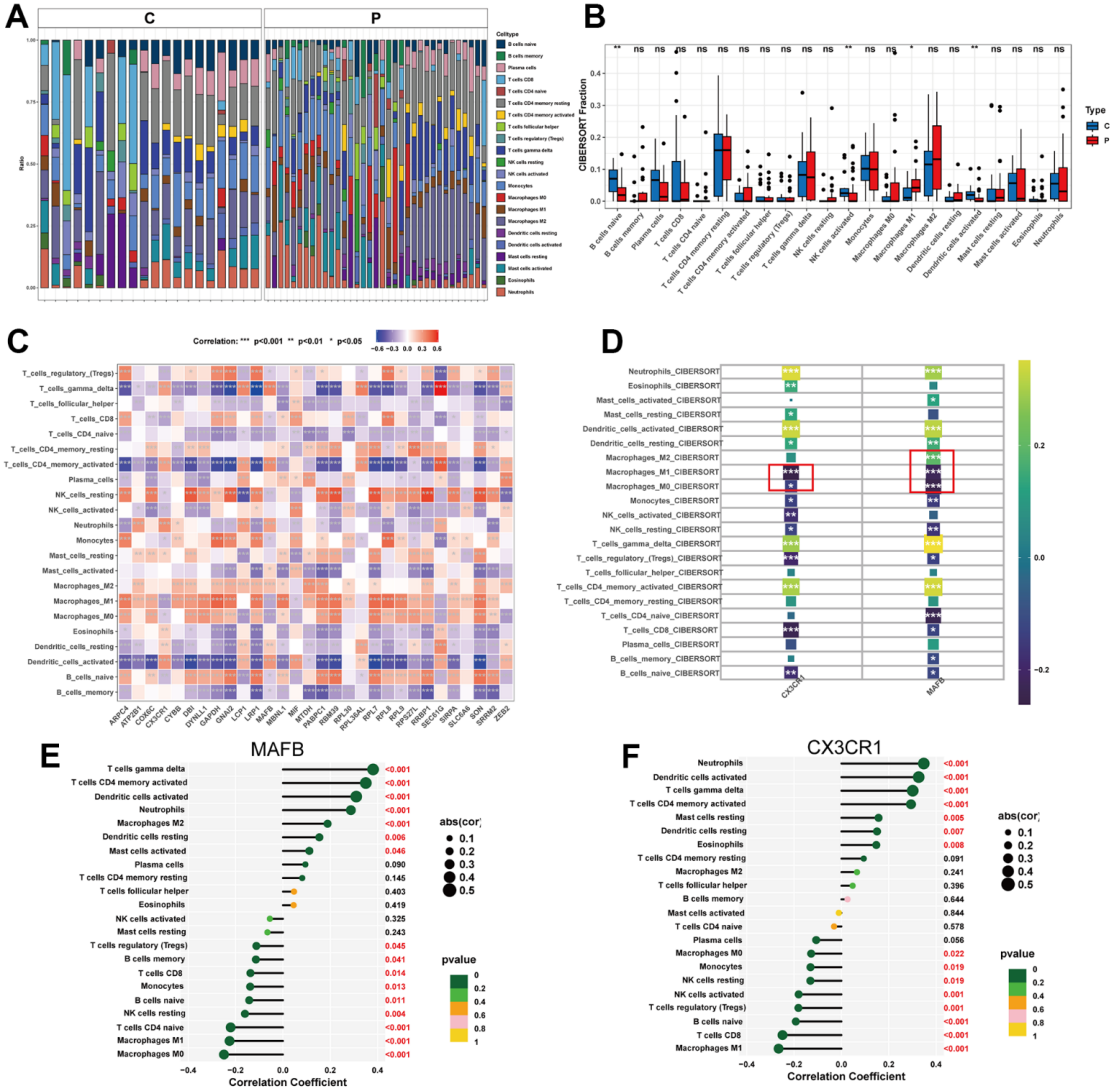
**Immune cell infiltration analysis.** (**A**) Heat map of the 22 immune cell subpopulations comparing NAFLD and normal samples. (**B**) Violin diagram illustrating the proportion of 22 different kinds of immune cells in NAFLD versus normal samples. (**C**) Heat map showing the correlation between 22 different kinds of immune cells and potential feature genes. The size of the colored squares indicates the connection’s strength; red indicates a positive correlation, while blue indicates a negative correlation. (**D**) Correlation between immune cells and optimal feature genes. (**E**) Correlation between MAFB and infiltrating immune cells. (**F**) Correlation between CX3CR1 and infiltrating immune cells. Correlation strength is proportional to the size of the dots. The color of the dots indicates the P-value. *P < 0.05, **P < 0.01, ***P < 0.001, ns, no significant difference.

In order to comprehensively assess immune cell profiles in normal and NAFLD samples, immune infiltration analysis was conducted. According to the results of CIBERSORT, there were significant differences in immune cell infiltration levels between NAFLD and normal samples (Figure 8A). NAFLD samples had a higher proportion of M1 macrophages, but relatively lower proportions of B cells, NK cells, and Dendritic cells than normal samples did (Figure 8B). The correlation heatmap demonstrated close associations between the potential feature genes and various immune cells (Figure 8C). We also performed separate analyses of the relationship between the optimal feature genes and immune cells (Figure 8D). The results showed that MAFB displayed a positive correlation with M2 macrophages, but negatively correlated with M0 and M1 macrophages (Figure 8E, Supplementary Figure 3A–3C). CX3CR1 showed a negative correlation with M0 and M1 macrophages (Figure 8F and Supplementary Figure 3D, 3E).

### The ssGSEA and GSEA analysis

**Figure 9:**
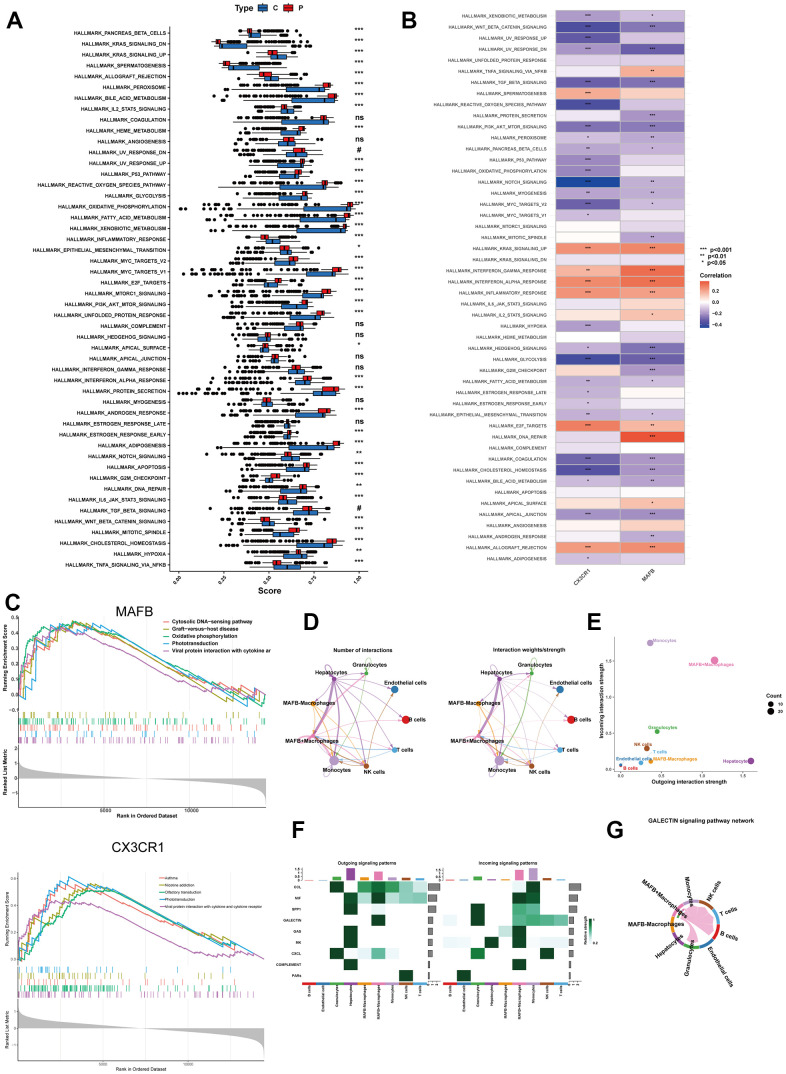
**Enrichment analyses by ssGSEA and single-gene GSEA.** (**A**) The specific distribution of 50 hallmark gene sets in the NAFLD group and control group samples. (**B**) The correlation analysis of 50 hallmark gene sets and 2 top feature genes. (**C**) Gene sets enrichment analysis (GSEA) identifies top five signaling pathways that are significantly enriched in the high expression of MAFB or CX3CR1. (**D**) Circle plot showing the communication strength between interacting cells. (**E**) Scatter plot indicating the incoming and outgoing interaction strength of the cells. (**F**) The dot plot showing the comparison of outgoing and incoming signaling patterns. (**G**) The cell communication between MAFB+ macrophages and other cells in the GALECTIN signaling pathway. *P < 0.05, **P < 0.01, ***P < 0.001.

We compared the abundance differences of 50 hallmark gene sets between the NAFLD group and the control group using the single-sample Gene Set Enrichment Analysis (ssGSEA) algorithm. In Figure 9A, we presented the distribution of these 50 gene sets in the NAFLD and control samples. We observed a significant upregulation of multiple gene sets in the NAFLD group compared to the control group. These upregulated gene sets include the peroxisome, bile acid metabolism, Heme metabolism, UV response up, P53 pathway, reactive oxygen species, glycolysis, oxidative phosphorylation, fat acid metabolism, xenobiotic metabolism, Myc targets v1, Myc targets v2, E2F targets, mTORC1 signaling, PI3K/AKT/mTOR Signaling, unfolded protein response, Interferon-alpha response, protein secretion, androgen response, estrogen response early, adipogenesis, apoptosis, G2M checkpoint, mitotic spindle, and cholesterol homeostasis. Additionally, we found that two top feature genes were positively correlated with the KRAS signaling up, interferon gamma response, interferon alpha response, inflammatory response, E2F targets and allograft rejection gene set (Figure 9B). For the single-gene GSEA analysis, the MAFB-activated pathway encompassed Cytosolic DNA-sensing pathway, Graft-versus-host disease, Oxidative phosphorylation, Phototransduction, and Viral protein interaction with cytokine and cytokine receptor (Figure 9C). The CX3CR1-activated pathway included Asthma, Nicotine Addiction, Olfactory transduction, Phototransduction, and Viral protein interaction with cytokine and cytokine receptor (Figure 9C).

Then, we investigated the role of MAFB+ macrophages in cell communication analysis. The results revealed that MAFB+ macrophages exhibited highly active signaling communication with other cells, while the MAFB- macrophages had rarely communicated with other cells (Figure 9D, 9E). The MAFB+ macrophages took part in more outgoing and incoming pathways than MAFB- macrophages (Figure 9F). Of note, the MAFB+ macrophages exhibited great strength in both incoming and outgoing patterns in MIF signaling pathway (Figure 9G).

### Validation of optimal feature genes in mouse module

**Figure 10:**
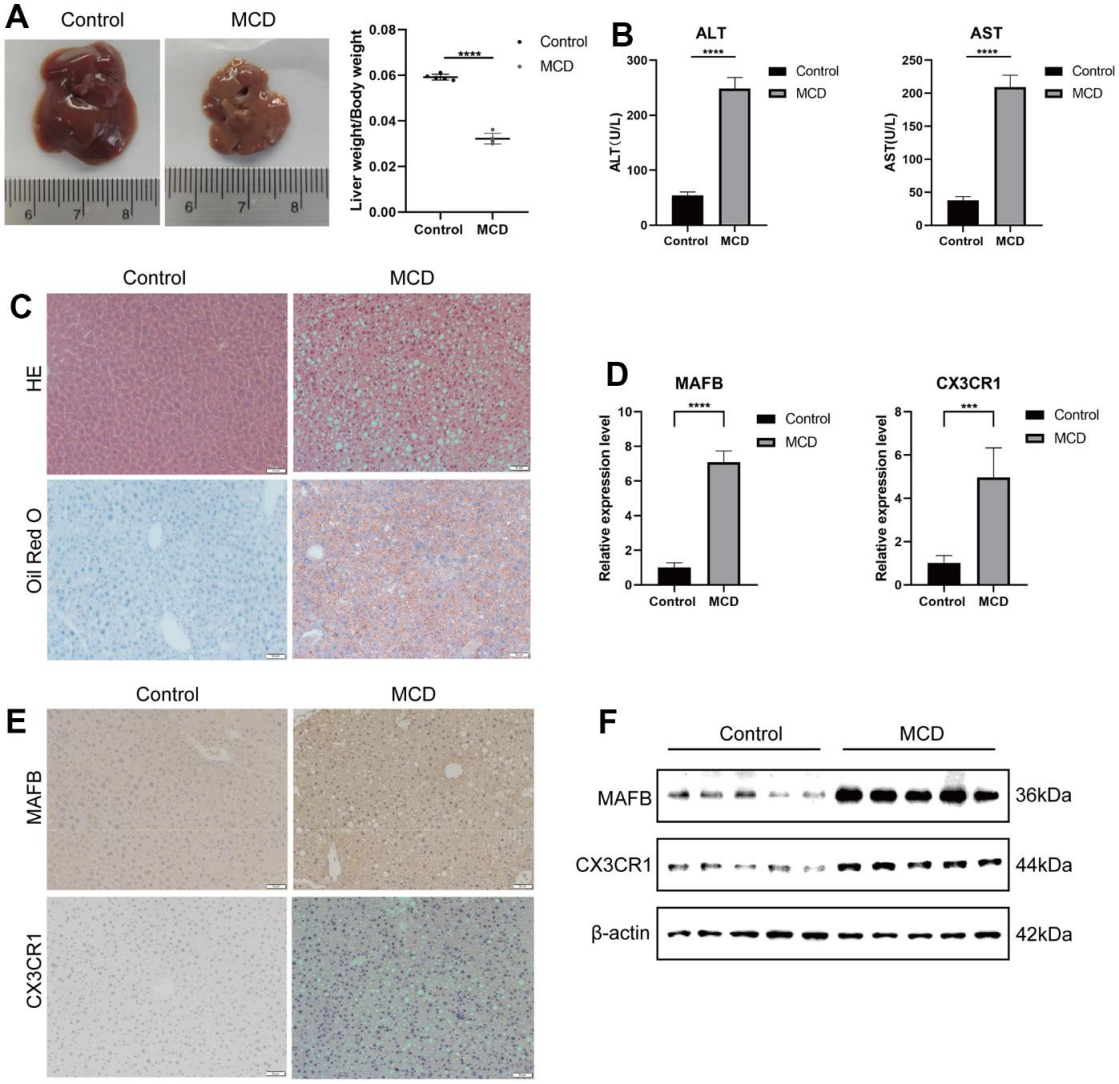
**Validation of optimal feature genes in mouse module.** (**A**) Fresh Livers and liver-to-body weight ratio in control and MCD mice. (**B**) The serum ALT and AST levels on control and MCD mice. (**C**) HE staining and Oil Red O staining of liver sections from mice fed on control or MCD-diet. (**D**) The relative expressions of MAFB and CX3CR1 were validated by RT-qPCR. (**E**) MAFB and CX3CR1 expression in liver tissues of control and MCD mice was detected by IHC. (**F**) MAFB and CX3CR1 expression in liver tissues of control and MCD mice was detected by WB. ***P < 0.001, ****P < 0.0001.

To assess the diagnostic value of the best characterized genes for NAFLD, we constructed the NAFLD mouse model and a normal mouse model. After four weeks of MCD feeding, MCD mice had decreased liver weight/body weight (Figure 10A), elevated serum ALT and AST (Figure 10B), enlarged vacuolization in liver cells (Figure 10C), which suggested that MCD mice had hepatitis injury. Furthermore, Oil Red O staining showed accumulating lipids in liver sections from mice fed on the MCD-diet (Figure 10C). After that, we used RT-qPCR (Figure 10D), IHC (Figure 10E), and western blot (Figure 10F) to detect the levels of MAFB and CX3CR1 in liver tissues of MCD and normal dietary mice (Figure 10F), which showed that MAFB and CX3CR1 were significantly overexpressed in the livers of MCD mice.

## DISCUSSION

The pathological course of NAFLD involves the role of genetic background and environmental factors and is associated with abnormalities in lipid metabolism, glucose metabolism, protein metabolism, and other aspects. This complexity increases the difficulty in understanding NAFLD [33]. Although non-invasive techniques such as ultrasound and alanine aminotransferase (ALT) can assist in the diagnosis of NAFLD, there are currently no established molecular markers that serve as key indicators for NAFLD [34]. According to prior studies, biomarkers at the genetic level can precisely determine the presence of disease and guide the development of clinical treatment strategies.

Therefore, we utilized multiple bioinformatics methods to explore the characteristic markers of NAFLD. Firstly, we derived the scRNA-seq data and the RNA-seq transcriptomic datasets of NAFLD from the GEO database for analysis. After processing the single-cell data, we found that the subtype 1 and subtype 7 macrophages were distinct in NASH mice. Cell-cell interaction analysis revealed close communication between NASH-macrophages and different cells. Then, we used the hdWGCNA algorithm to identify the modules most associated with NASH-macrophages, resulting in 40 genes representing the functionality of NASH-macrophages. After cross-correlated them with human transcriptome data, resulting in 30 potential characteristic genes. Through immune infiltration analysis, we also found their close association with various immune cells, particularly macrophages. Then, we combined three machine learning algorithms, LASSO regression, SVM, and Random Forest, and finally identified two optimal feature genes (MAFB and CX3CR1) closely associated with the diagnosis or progression of the disease. In addition, the ROC results show that both CX3CR1 and MAFB genes have elevated diagnostic performance for NAFLD on both training and validation sets. Functional enrichment analysis using GSVA and single gene GSEA was performed on the optimal feature genes. Immune infiltration analysis revealed the optimal feature genes were statistically associated with macrophage infiltration, consistent with the module correlations obtained from hdWGCNA. Moreover, we constructed an NAFLD mouse model to further validate the expression of MAFB and CX3CR1. In conclusion, our study identified a NAFLD-associated macrophage subpopulation and the NAFLD feature gene.

Previous studies have found that an increase in portal vein macrophages is one of the earliest changes observed in liver biopsy specimens of patients with fatty liver disease [35]. In a high-fat diet-induced mouse model of fatty liver disease, the release of interleukin-1 beta (IL-1β) by KCs promotes hepatic steatosis by inhibiting peroxisome proliferator-activated receptor alpha (PPARα) activity in hepatocytes [7]. It has been found that under hepatic lipotoxic conditions, the release of inflammatory factors by hepatocytes induces the infiltration of macrophages into the liver [36]. Several studies have reported that macrophages can interact with hepatic stellate cells through cytokines and chemokines during the progression of NAFLD, promoting collagen deposition and fibrosis, ultimately leading to liver fibrosis and cirrhosis [37, 38]. Activated macrophages also participate in the regulation of fatty acid metabolism and lipid deposition in NAFLD [39]. This is consistent with our findings of activation of lipid metabolism signaling pathways in ssGSEA. In summary, macrophages play a crucial role in the progression of NAFLD and warrant additional investigation.

Our pseudo-temporal analysis describes macrophage development and indicates that NASH-macrophages are predominantly concentrated in the late stages of macrophage differentiation. Previous literature reports have shown that late-stage differentiated macrophages express additional surface molecules, including various tissue-specific surface markers and receptors [40]. This is consistent with our analysis, as some receptor-related genes, such as CX3CR1, Gani2, and Son, are expressed in the late stages of macrophage differentiation. In the transcription factor prediction analysis, it is clear that the RXRa is greatly expressed in the NASH group. Retinoid X receptor alpha (RXRA) is a member of the nuclear receptor superfamily that participates in lipid, glucose, energy, and hormone metabolism. RXRA can accelerate lipid accumulation by regulating the transcription of target genes that promote lipid accumulation [41]. Furthermore, RXRA may potentially increase people’s risk of developing Alzheimer’s disease by affecting brain cholesterol metabolism [42]. These analyses suggest that NASH-macrophages and their associated genes play a particularly prominent role in the progression of NAFLD.

Of the 30 genes that could be characterized, we identified the 2 most closely related to NAFLD. The function of the transcription factor V-maf musculoaponeurotic fibrosarcoma oncogene homologue B (MAFB) in the development of NAFLD has been extensively studied. As early as 2000, Kelly et al. indicated that overexpression of MAFB resulted in differentiation of chicken bone marrow primitive cells to macrophages [43]. Several studies showed that MAFB function is indispensable in disease-associated macrophages [44, 45]. Recently, Cuevas VD et al. found that MAFB is essential to the acquisition of anti-inflammatory transcriptional and functional characteristics in human macrophages [46]. This phenomenon was verified by Basile et al., who suggested that MAFB-mediated macrophage differentiation is involved in intrinsic repair after acute kidney injury [47]. CX3CR1 is a gene that encodes a chemokine receptor involved in immune and inflammatory processes. Sutti et al. found that inflammatory dendritic cells expressing CX3CR1 cells promote the development of nonalcoholic steatohepatitis [48]. The research conducted by Ni Y et al. demonstrated a significant upregulation of CX3CR1 expression in liver macrophages within NASH mice, as compared to their counterparts in normal mice [49]. Hence, we contend that the optimal feature genes play a crucial role in the initiation of NAFLD.

The methionine and choline deficiency (MCD) diet-induced NAFLD model is one of the most classical models. Its principle involves the deficiency of methionine and choline, which hinders the necessary processes of beta-oxidation and very low-density lipoprotein synthesis [50]. Mice fed with the MCD diet exhibit characteristics such as weight loss, decreased levels of serum triglycerides (TG), and reduced liver weight-to-body weight ratio, which are contrary to the phenotype of human fatty liver disease [51]. Pathological validation of the model commonly involves the use of HE and oil red O staining. Through validation using the MCD mouse model, we observed increased expression of MAFB and CX3CR1 at the RNA and protein levels in liver tissue, further confirming their accuracy in the diagnosis of NAFLD.

All in all, we first identified a special cluster of macrophages playing an important role in NAFLD. Secondly, we reported for the first time that MAFB and CX3CR1 are characteristic genes of NAFLD. Immune infiltration analysis validated the relationship between these two genes and macrophages. Additionally, the diagnostic performance of these two genes was confirmed through the construction of an animal model. However, our study also has limitations. On one hand, we did not thoroughly explore the expression patterns of these two genes in macrophages. Furthermore, we lacked a large clinical cohort to explore the diagnostic value of these characteristic genes. In summary, our findings may bring new hope for the early diagnosis of NAFLD. Further research on the specific mechanisms and regulatory pathways of MAFB and CX3CR1 mediated by macrophages in NAFLD development will help enhance our understanding of the pathogenesis of NAFLD and potentially provide new targets for its treatment.

## CONCLUSIONS

In conclusion, our study demonstrates a diagnostic model that can be applied to NAFLD. These findings will help to better reveal the role of macrophages in the progression of NAFLD. Meanwhile, the NAFLD characteristic genes identified in this study, especially MAFB and CX3CR1, may shed new light on the clinical development of effective diagnosis and treatment of NAFLD.

## Supplementary Material

Supplementary Figures

Supplementary Table 1
