# Supplementary material for: Identification and validation of key biomarkers associated with macrophages in nonalcoholic fatty liver disease based on hdWGCNA and machine learning
Source: Aging (Albany NY). 2023 Dec 21;15(24):15451–72. doi: 10.18632/aging.205374 (PMC10781485; doi:10.18632/aging.205374)
Supplement: Supplementary Figures [file aging-15-205374-s001.pdf]

SUPPLEMENTARY FIGURES

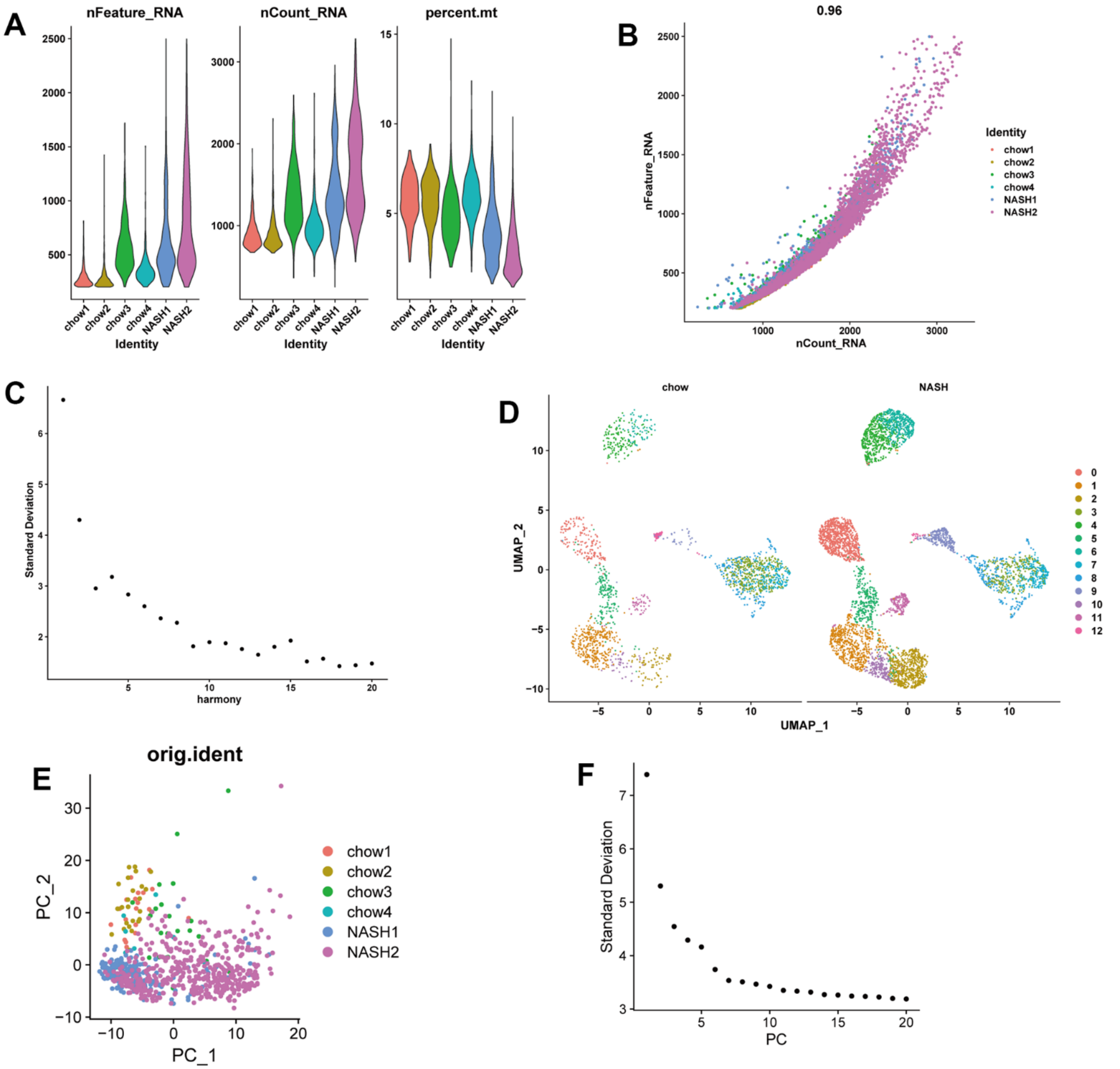

Supplementary Figure 1. Preprocessing of scRNA-seq data. (**A**) The features, counts, and percentages of mitochondrial genes in each of the analyzed samples. (**B**) The Scatter Plot demonstrates the correlation between cell counts and intracellular gene counts following standardization. (**C**) Selecting the principal components (PCs) for further analysis. (**D**) UMAP plot visualizes the clusters in control and NASH mouse livers. (**E**) PCA dimension reduction of macrophages. (**F**) Selecting the principal components (PCs) for further analysis.

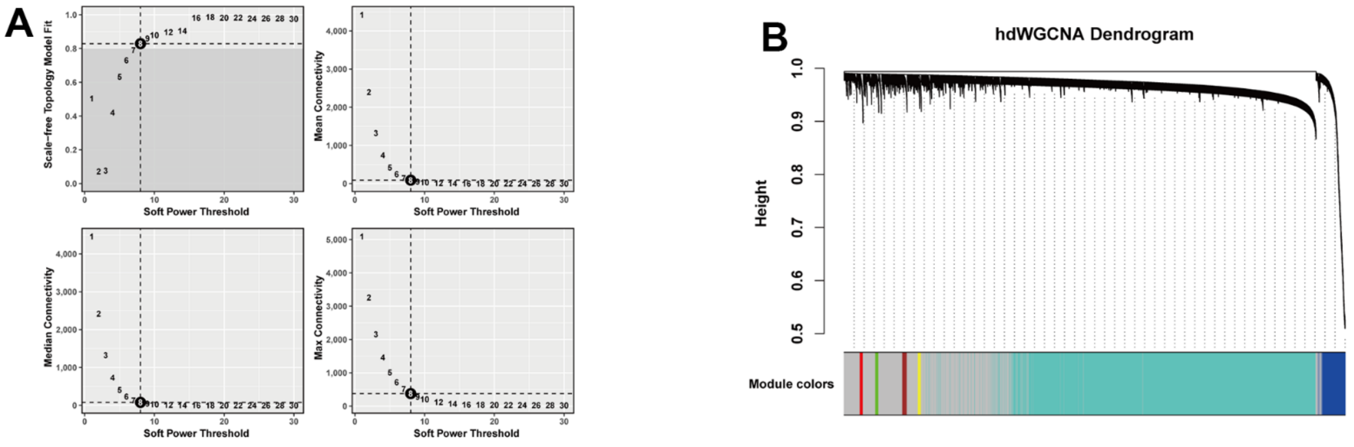

Supplementary Figure 2. The process of hdWGCNA. (**A**) Scale-free fit index and mean connectivity plot for various soft threshold powers. (**B**) The hdWGCNA dendrogram of macrophages.

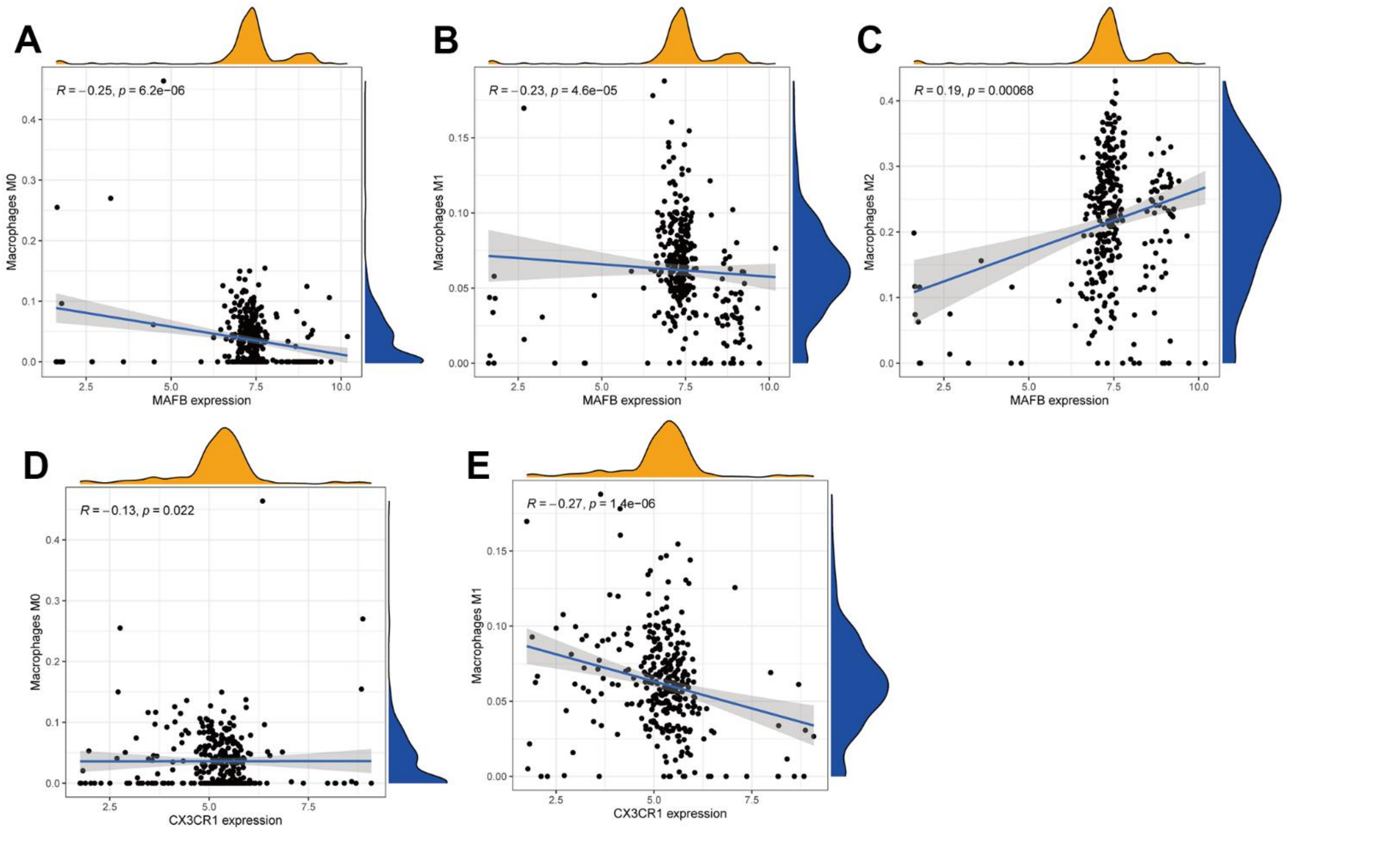

Supplementary Figure 3. Immune cell infiltration analysis. Correlation between MAFB and M0 macrophages (**A**), M1 macrophages (**B**), and M2 macrophages (**C**); Correlation between CX3CR1 and M0 macrophages (**D**) and M1 macrophages (**E**).
