# Supplementary material for: Identification and validation of key biomarkers associated with macrophages in nonalcoholic fatty liver disease based on hdWGCNA and machine learning
Source: Aging (Albany NY). 2023 Dec 21;15(24):15451–72. doi: 10.18632/aging.205374 (PMC10781485; doi:10.18632/aging.205374)
Supplement: Supplementary Table 1 [file aging-15-205374-s002.pdf]

SUPPLEMENTARY TABLE

Supplementary Table 1. The primers designed for RT-qPCR.

Gene	Primer
Mus-Actin (Forward)	TCCTTCCTGGGCATGGAG
Mus-Actin (Reverse)	AGGAGGAGCAATGATCTTGATCTT
Mus-MAFB (Forward)	TGAATTTGCTGGCACTGCTG
Mus-MAFB (Reverse)	AAGCACCATGCGGTTCATACA
Mus-CX3CR1 (Forward)	CAGCATCGACCGGTACCTT
Mus-CX3CR1 (Reverse)	GCTGCACTGTCCGGTTGTT
